# Obtaining and Characterization of New Materials, Volume V

**DOI:** 10.3390/ma19040819

**Published:** 2026-02-21

**Authors:** Andrei Victor Sandu

**Affiliations:** 1 Faculty of Materials Science and Engineering, “Gheorghe Asachi” Technical University of Iasi, 41 “D. Mangeron” Street, 700050 Iasi, Romania; andrei-victor.sandu@academic.tuiasi.ro; 2 Academy of Romanian Scientists, 54 Splaiul Independentei St., Sect. 5, 050094 Bucharest, Romania; 3 Romanian Inventors Forum, Sf. P. Movila 3, 700089 Iasi, Romania

Volume V of the Special Issue “Obtaining and Characterization of New Materials” continues to provide a multidisciplinary venue for reporting cutting-edge breakthroughs in materials science and engineering, building on the success of earlier volumes. This volume’s contributions focus on the synthesis, processing, characterization, and application-oriented understanding of novel materials at various scales, from micro to macro.

The studies in this Special Issue emphasize the importance of advanced processing approaches combined with modern characterization tools for customizing structure–property correlations. The topics include lightweight and porous materials, functional surfaces and coatings, polymer and composite systems, advanced alloys, cementitious materials, environmental remediation materials, and energy-related materials.

The contributions to this volume represent the growing demand for innovative, sustainable, and multifunctional materials. The emphasis is on understanding structure–property–performance correlations using current characterization techniques such as advanced microscopy, spectroscopy, thermal analysis, mechanical testing, and functional assessment. This understanding is critical for directing material selection, optimizing processing pathways, and ensuring dependable deployment in engineering, biomedical, environmental, and energy-related applications.

Despite significant advances in material development, a number of knowledge gaps remain across a wide range of application areas. These include limitations in the transferability of laboratory-scale processing routes to real-world service conditions, a lack of understanding of long-term durability and aging mechanisms, incomplete multiscale structure–property correlations, and challenges with sustainability, recyclability, and circular economy integration. In this context, the current Special Issue aims to fill these gaps by bringing together studies that not only report on novel materials and processes, but also provide mechanistic insight, comparative evaluation, and application-oriented validation.

This volume contains research on porous metallic foams, activated carbons, asphalt and road materials, surface engineering technologies, polymeric and biomedical materials, metallic alloys, sustainable building materials, advanced manufacturing methods, and phase change materials for thermal energy storage.

Through this integrative approach, the Special Issue responds directly to the need for materials solutions that are not only high-performing, but also durable, scalable, and environmentally responsible.

This volume’s thematic organization reflects the current evolution of materials science toward convergence in functionality, sustainability, and advanced manufacturing. The sections that follow show how diverse material classes—ranging from porous metals and carbons to polymers, ceramics, composites, and multifunctional inorganic systems—can be engineered through controlled processing and validated through extensive characterization to meet specific technological demands.

## 1. Porous, Carbon-Based, and Environmentally Oriented Materials

Thalmaier et al. showed that press-and-sinter processing can be used to make syntactic magnesium foams that are floatable, have low densities, and are strong enough for functional shaping [1]. The materials made it possible for Marangoni-driven propulsion through surface tension gradients, which led to high transient speeds and showed that they could be used for microscale transport and mass transfer [2,3,4].

The following research [5] involved oxidative stabilization and steam activation to create activated carbons generated from petroleum pitch that have customized micro-mesoporous structures. The high surface areas and increased mesoporosity made it easy to get rid of siloxanes and H_2_S, which shows how important pore size distribution is in gas purification [6,7,8].

Kuo et al. used phosphoric acid to activate oil-containing sludge, which made mesoporous, magnetic carbon composites [9]. The carbons that come out of this process have small surface areas and are easy to recover magnetically, which makes them good for use as adsorbents or catalytic supports made from industrial waste streams [10,11,12].

Nartowska et al. [13] looked at how Zn and Cu move through bentonites and found important links between ion mobility, clay structure, and the amount of unfrozen water. The study elucidates the efficacy of bentonite in environmental remediation, especially under subzero conditions [14,15,16].

## 2. Asphalt, Road Materials, and Thermo-Rheological Performance

Hou et al. investigated natural and laboratory PAV aging of asphalt binders, finding that field aging in cold-arid locations causes more severe loss of low-temperature performance [17]. The findings highlight basic variations in aging mechanisms and the limits of current accelerated aging techniques [18,19,20].

Liu et al. created nano-TiO2-modified twofold phase change asphalt with paraffin-based composite PCMs [21]. Increased thermal conductivity and better phase transition behavior minimized temperature extremes while keeping acceptable rheological characteristics [22,23,24].

Other important studies looked into biochar-modified asphalt binders made from oat hulls and found that they were better at resisting rutting, performing well under stress, and aging [25]. The research advocates for biochar as a sustainable modifier in pavement engineering [26,27].

## 3. Surface Engineering, Thin Films, and Coatings

Iatan et al. adjusted pulsed laser cladding settings to recondition Ni-Al-Bronze marine propellers [28]. Defect-free CuNi coatings with increased hardness and corrosion resistance were created, bolstering laser cladding as a sustainable repair approach [29,30,31].

Another study looked into how the temperature of the substrate affects KBr thin films made by thermal evaporation [32]. Researchers found that the structure and optical properties do not always change in a straight line, which means there is a good range of processing conditions for infrared and cryogenic applications [33,34,35].

Mladenović et al. found that grain refinement and grain boundary density significantly affect adhesion performance in electrodeposited copper films [36,37,38].

Also, Laptoiu et al. enhanced hydroxyapatite deposition on titanium alloys with pulsed electrical discharge, resulting in highly adherent bioactive layers suited for implant surface biofunctionalization [39,40,41,42,43].

## 4. Metals, Alloys, and Functional Inorganic Materials

Prica et al. produced Cu-Al-Ni nanocrystalline compacts via mechanical alloying and spark plasma sintering, displaying controlled phase evolution and densification appropriate for shape memory applications [44,45,46,47].

The effects of protracted milling on Fe-Mn-Sn Heusler alloys and discovered stable nanocrystalline A2 structures as well as milling-induced variations in electrical resistivity was studied by Popa et al. [48,49,50].

Tian et al. employed synchrotron X-ray fluorescence microscopy to study Fe segregation during the solidification of Al-Zn-Mg-Si alloys, revealing a peritectic process required for intermetallic production [51,52].

Tomaszewicz et al. created Nd3+-doped scheelite-type molybdato-tungstates that exhibited exceptional thermal stability and multifunctional optical and magnetic properties [53,54,55].

Using advanced spectroscopy and modeling, Bakradze et al. revealed the structure of polaronic centers in proton-intercalated scheelite-type tungstates, which advances our understanding of charge localization in oxides [56,57,58].

## 5. Polymers, Composites, and Biomedical Materials

Enhanced dispersion of graphene nanoplatelets within an ABS matrix allowed for the creation of nanocomposite films with better thermal stability, attractive dielectric characteristics, and excellent electromagnetic interference shielding performance [59,60].

Moisture-resistant amorphous solid dispersions were developed for a poorly soluble medicinal drug, highlighting the importance of careful polymer selection in guaranteeing long-term physical stability and prolonged dissolving behavior [61,62].

Electrospun PLA fiber systems combined with cyanoacrylate-based tissue adhesives were created for hemostatic applications, resulting in quick blood coagulation and robust tissue adherence, as demonstrated by in vivo testing [63,64,65].

## 6. Sustainable Cementitious Materials, Manufacturing, and Metrology

At low doses, partial replacement of Portland cement with high-iron-content sludge and sludge ash has been demonstrated to be viable, while retaining both mechanical performance and environmental safety [66,67,68].

Gu et al. also demonstrated that calcined sewage sludge ash may efficiently substitute for calcined clay in limestone calcined clay cement (LC3) systems, allowing heavy metals to be immobilized within the cementitious matrix [69,70].

Incorporating TiO_2_ nanoparticles into cementitious composites results in self-cleaning and biocidal characteristics. The type of the aggregates determines microbial resistance [71,72].

Industrial waste was valorized by converting circulating fluidized bed boiler ash into inorganic fibers with tensile qualities similar to basalt fibers [73].

Li et al. created an additively built metamaterial-based Luneburg lens antenna capable of broadband operation and wide-angle beam scanning, indicating a high potential for next-generation communication systems [74,75].

Jaskólski et al. [76] found that altering the shape of ceramic grinding wheels used in dual-tool grinding heads enhanced surface quality on components with complex geometries.

Fractal-based techniques to surface characterization were compared and found to be suitable for consistent multiscale analysis of manufactured surface topographies [77,78].

A thorough thermophysical analysis of guanidinium methanesulfonate revealed its low volatility, thermal stability, and appropriateness as a safe and efficient phase transition material for thermal energy storage applications [79].

In conclusion, the fifth edition of this Special Issue focuses on the breadth and depth of current materials research, emphasizing the role of advanced processing routes and comprehensive characterization in the development of sustainable and high-performance materials. The collected contributions show that significant progress in materials science is increasingly dependent on the integration of synthesis, multiscale characterization, and functional validation.

Beyond documenting recent advances, this volume suggests several key directions for future research. These include the need for better modeling and data-driven approaches to predict structure–property relationships, further research into long-term stability and degradation mechanisms, and the development of scalable and environmentally friendly processing technologies. Furthermore, closer alignment of experimental studies and real-world service conditions is required to accelerate the transition of new materials from laboratory research to industrial and societal applications.

This Special Issue aims to stimulate further innovation and interdisciplinary collaboration in materials science and engineering by addressing existing knowledge gaps and identifying emerging research priorities, thereby supporting the development of next-generation materials tailored for engineering, environmental, biomedical, and energy-related challenges.
